# Effects of elevated CO_2_ on biomass and fungi associated with two ecotypes of ragweed (*Ambrosia artemisiifolia* L.)

**DOI:** 10.3389/fpls.2014.00500

**Published:** 2014-09-26

**Authors:** G. Brett Runion, Stephen A. Prior, Andrew J. Price, J. Scott McElroy, H. Allen Torbert

**Affiliations:** ^1^U.S. Department of Agriculture, Agricultural Research Service, National Soil Dynamics LaboratoryAuburn, AL, USA; ^2^Department of Crop, Soil and Environmental Sciences, Auburn UniversityAuburn, AL, USA

**Keywords:** *Ambrosia artemisiifolia*, common ragweed, elevated CO_2_, fungi, glyphosate, herbicide resistance

## Abstract

Herbicide resistant weed populations have developed due to the repeated application of herbicides. Elevated concentrations of atmospheric CO_2_ can have positive effects on weed growth, but how rising CO_2_ might affect herbicide resistant weeds is not known. Ragweed (*Ambrosia artemisiifolia* L.) ecotypes known to be resistant or susceptible to glyphosate herbicide were exposed to either ambient or elevated (ambient +200 μ mol mol^−1^) concentrations of CO_2_ in open top chambers. Plants were harvested following 8 weeks of CO_2_ exposure; at this time, they had begun to exhibit disease symptoms including spots on leaves and stems. Elevated CO_2_ significantly increased top, root, and total plant biomass. Also, glyphosate resistant plants had significantly greater top, root, and total biomass than plants susceptible to the herbicide. There were no significant CO_2_ by ecotype interactions. Fungi from 13 genera were associated with ragweed, several of which can be either pathogens (i.e., *Alternaria*, *Fusarium*, *Rhizoctonia*), aiding the decline in health of the ragweed plants, or saprophytes existing on dead plant tissues. The common foliar disease powdery mildew was significantly higher on susceptible compared with resistant ragweed. Susceptible plants also showed an increased frequency of *Rhizoctonia* on leaves and *Alternaria* on stems; however, *Fusarium* occurred more frequently on resistant ragweed leaves. Fungi were not affected by CO_2_ concentration or its interaction with ecotype. This study reports the first information on the effects of elevated CO_2_ on growth of herbicide resistant weeds. This is also the first study examining the impact of herbicide resistance and elevated CO_2_ on fungi associated with weeds. What effects herbicide resistance might have on plant diseases and how rising atmospheric CO_2_ might impact these effects needs to be addressed, not only with important weeds but also with crops.

## Introduction

Most plants exhibit a positive growth response to elevated CO_2_ due to increased photosynthesis and/or nutrient use efficiency (Rogers et al., 1983b; Amthor, 1995; Kimball et al., 2002) and weeds are no exception (Patterson et al., 1984; Bazzaz et al., 1989). In fact, Ziska (2003) reported that the CO_2_-induced growth stimulation of several invasive weeds was greater than for any previously examined plant species. We found that six important southeastern U.S. weeds had positive growth responses to elevated CO_2_ suggesting they may become more problematic as atmospheric CO_2_ continues to rise (Rogers et al., 2008; Runion et al., 2008; Smith et al., 2008; Price et al., 2009). This response to rising atmospheric CO_2_ can have important implications for weed control.

Evidence suggests that elevated CO_2_ may increase herbicide tolerance in some weeds (Ziska et al., 1999). This tolerance may be due to a herbicide dilution effect caused by increased growth. Also, changes in weed morphology (e.g., increased leaf thickness) and physiology (e.g., decreased stomatal conductance) can alter herbicide uptake, translocation, and overall efficacy (Ziska and Teasdale, 2000). This raises concerns about how weed control strategies in agriculture production systems will change in a future, higher CO_2_ world.

Weed control strategies will be further complicated by the fact that herbicide resistant weed populations are being identified (Dill et al., 2008; Price et al., 2011). Repeated application of herbicides, particularly those with the same modes of action, results in the development of weed populations resistant to herbicidal control. To date, approximately 235 different weed species have developed resistance to 22 of the 25 known herbicide sites of action and to 155 different herbicides; herbicide resistant weeds have been reported in 82 crops in 65 countries (Heap, 2014).

When weed populations develop herbicide resistance, other traits might also be selected for or against. For example, herbicide resistant weeds may have an altered response to microbes, resulting in increases or decreases in disease. It is known that effects of elevated CO_2_ on plant disease can vary depending upon the host, microbe, and environment (Ziska and Runion, 2007). Research has shown that elevated CO_2_ can increase (Chakraborty et al., 2000; Eastburn et al., 2010; McElrone et al., 2010), decrease (McElrone et al., 2005; Strengbom and Reich, 2006; Eastburn et al., 2010; Runion et al., 2010), or have no affect (Tiedemann and Firsching, 2000; Karnosky et al., 2002; Ferrocino et al., 2013; Oehme et al., 2013) on plant diseases. Bebber et al. (2014) recently emphasized the importance of understanding how shifts in the distribution of crops pests and diseases (particularly fungi) threatens global food security in a changing climate. However, how rising atmospheric CO_2_ might affect plant diseases on emerging herbicide resistant weed populations has not been addressed.

The initial objective of this study was to examine the growth response of glyphosate susceptible and resistant populations of common ragweed (*Ambrosia artemisiifolia* L.) to elevated CO_2_. Given that plants displayed symptoms of disease infection at harvest, a secondary objective was added to examine how CO_2_ and ragweed ecotype affected fungal frequency.

## Materials and methods

A common ragweed ecotype resistant to glyphosate (N-[phosphonomethyl] glycine) was identified in Madison County, AL, and a ragweed ecotype susceptible to the herbicide was identified at the Alabama Agricultural Experiment Station's E.V. Smith Research Center in Macon County, AL. Seed from these two ragweed ecotypes were collected from these respective areas and germinated in soil flats in a greenhouse at the USDA-ARS National Soil Dynamics Laboratory, Auburn, AL. Soon after emergence, plants of each ecotype were transplanted into trade gallon containers filled with a general purpose growth medium (PRO-MIX Bx, Premier Horticulture Inc., Quakertown, PA 18951) at one plant per container. Plants were held in the greenhouse for 3 weeks where they received water daily.

Thirty-two containers of each ecotype were visually ranked according to plant size, and placed into four groups of eight containers each, representing the largest eight first in declining order down to the smallest eight. The eight containers from each of the four ranked groups for each ragweed ecotype were randomly assigned to either an ambient or an elevated CO_2_ open-top field chamber (OTC) in each of four blocks. Therefore, the first block was assigned the largest eight plants used in the study and the fourth (last) block was assigned the smallest eight plants. In this way, any effects of initial plant size would be incorporated into the block effect.

The study was conducted at the soil bin facilities at the USDA-ARS National Soil Dynamics Laboratory, Auburn, AL. The bin used for the experiment is 6 m wide and 76 m long and has been modified by installing a geomembrane liner (20 mil) and gravel drain system to ensure a good working surface and drainage for container studies. Open top chambers (Rogers et al., 1983a), encompassing 7.3 m^2^ of ground surface area, were used to continuously (24 h per day) monitor ambient CO_2_ and deliver an elevated CO_2_ concentration of ambient plus 200 μ mol mol^−1^ using a previously described delivery and monitoring system (Mitchell et al., 1995). The bin was divided into four blocks and each CO_2_ treatment was randomly assigned to one OTC within each block. The experiment was conducted as a split plot design with CO_2_ level being the main plot factor and ecotype being the split plot factor with the four blocks occurring along the length of the soil bin.

Plants were placed in the OTCs on 9 September, 2013. All pots received water (~400 ml) two to three times per week depending on rainfall. Pots were fertilized upon placement in the OTCs and on weeks 2 and 6 of the study. Fertilization was accomplished using Miracle-Gro (15:30:15, N: P_2_O_5_: K; Scotts Products Inc., Marysville, OH) according to fertilizer manufacturer's recommendations. Liquid Sevin (0.126% Carbaryl; GardenTech, Palatine, IL) was sprayed on all plants during weeks 2, 3, and 7 to control white flies. In addition, granular Sevin (2% Carbaryl; GardenTech, Palatine, IL) was applied to soil in all pots during weeks 3 and 4 for general insect control. Plants were destructively harvested on 4 November, 2013, corresponding to 8 weeks of CO_2_ exposure. At harvest, it was noted that plants looked unhealthy, with spots on leaves and stems, and/or were beginning to senesce. This natural infection or colonization was a situation worth examining further.

Aboveground portions of plants in each container were harvested by severing the plant at the ground-line. Roots were separated from the growth medium using the sieve method (Bohm, 1979). Although this was initially intended as a short-term biomass study, the fact that plants looked unhealthy, with spots on leaves and stems, led us to examine them for possible associations with fungi. Nine to 12 sections (total), each ~1 cm in length, were cut from the four plants of each ecotype harvested from each OTC for each plant component part (leaves, stems, and roots) using forceps and scissors which were flame sterilized between each group's component parts. The remaining tissue from each plant, separated as aboveground and belowground, was placed in a brown paper bag, dried in a forced-air oven at 55°C to a constant weight, and dry weights were recorded. Total dry weight data, separated as to aboveground and belowground, were averaged for the four plants of each ecotype from each OTC prior to data analysis.

Each group of excised plant sections were placed into three plastic petri dishes (100 × 15 mm) containing a piece of sterile filter paper (Whatman #42) moistened with sterile water (Barnstead Nanopure Diamond with a 0.2 μ m final filter, Lake Balboa, CA). Petri dishes were sealed with Parafilm and placed in a dark incubator at room temperature (25°C). All petri dishes were checked daily for the presence of fungi growing on or from the plant component sections. Leaf sections tended to demonstrate fungal growth more rapidly than either stems or roots and, after 1 week of incubation, all petri dishes containing leaf sections were examined for fungi under a dissecting microscope (Olympus model SZH10 Research Stereoscope). Fungi observed under the dissecting microscope were carefully excised, placed onto standard microscope slides, examined under a compound microscope (Nikon Optiphot), and identified to genus with the aid of taxonomic keys (Barnett and Hunter, 1972; Hanlin, 1990). Fungi from stem and root sections were similarly processed after 10 and 14 days of incubation, respectively. Fungal data recorded were the total number of plant component sections in the three petri dishes and the total number of sections on which a particular fungal genus was observed. These data were then used to calculate a percentage of sections yielding a fungal genus [(sections yielding/total sections) × 100]. These percent data were square root transformed prior to data analysis.

Data analysis was conducted using the mixed model procedures (Proc Mixed) of the Statistical Analysis System (Littell et al., 1996). Error terms appropriate to the split plot design were used to test the significance of CO_2_ concentration and ecotype. In all cases, differences were considered significant at the α ≤ 0.05 and trends were recognized at 0.05 < α ≤ 0.10.

## Results and discussion

Plants growing under elevated atmospheric CO_2_ had significantly greater top, root, and total biomass compared to plants growing under ambient CO_2_ (Table 1). In addition, plants resistant to glyphosate had significantly greater top, root, and total biomass than plants susceptible to the herbicide (Table 1). Further, although the CO_2_ by ecotype interaction was not significant, it is interesting to note that resistant plants tended to have greater biomass regardless of CO_2_ concentration but elevated CO_2_ tended to increase biomass only in resistant plants (Table 1).

**Table 1:** **The response of two ecotypes (R = Resistant; S = Susceptible to glyphosate) of common ragweed plant component part dry weight to ambient (A = 375 μ mol mol^−1^) and elevated (E = ambient + 200 μ mol mol^−1^) CO_2_**.

**CO_**2**_ Level**	**Ecotype**	**Above**	**Root**	**Total plant**
A	R	1.05^†^	0.12	1.17
E	R	1.42	0.17	1.59
A	S	0.39	0.05	0.44
E	S	0.47	0.08	0.55
**Statistics**		***P*-values**	***P*-values**	***P*-values**
CO_2_		0.046	0.029	0.033
Ecotype		<0.001	0.001	<0.001
CO_2_ × Ecotype		0.169	0.555	0.173
A vs. E in R		0.024	0.049	0.020
A vs. E in S		0.574	0.193	0.480
R vs. S in A		0.001	0.017	<0.001
R vs. S in E		<0.001	0.004	<0.001

Means with associated separation statistics are shown.

† Data are plant dry weight in grams.

Most plants, assuming other factors (e.g., water and nutrients) are not limiting, exhibit a positive growth response to elevated CO_2_ due to increased photosynthesis and/or nutrient use efficiency (Rogers et al., 1983b; Amthor, 1995; Kimball et al., 2002). Weeds are no exception to this general rule (Patterson et al., 1984; Bazzaz et al., 1989). Six weeds important in the southeastern U.S. all had positive growth responses suggesting they may become more problematic under rising atmospheric CO_2_ (Rogers et al., 2008; Runion et al., 2008; Smith et al., 2008; Price et al., 2009). Growth response to elevated CO_2_ in the current study was consistent with that reported for C_3_ plants (33–40% increase; Kimball, 1983). However, Ziska (2003) reported that the CO_2_-induced growth stimulation of six invasive weeds was greater (~3×) than for any previously examined species and that this will have important implications for control.

Exacerbating the problem of control, evidence suggests that elevated CO_2_ may increase herbicide tolerance in some weeds (Ziska et al., 1999; Ziska and Teasdale, 2000). This leads to concerns regarding efficacy of herbicidal control strategies in agriculture production systems. Further, herbicide resistant weed populations are being identified (Dill et al., 2008; Price et al., 2011) due to the repeated application of herbicides with the same modes of action. To date, approximately 235 different weed species have developed resistance to 22 of the 25 known herbicide sites of action and to 155 different herbicides; herbicide resistant weeds have been reported in 82 crops in 65 countries (Heap, 2014). What other genetic alterations might accompany this developed resistance and how they might affect interactions with plant diseases are unknown. Further, how rising atmospheric CO_2_ might affect this resistance and its associated effects on plant diseases has not been addressed.

We observed fungi from a total of 13 genera associated with ragweed plants. Leaves yielded a greater number of fungal genera (8) than did stems or roots (5 each). Several of the fungi found on leaves are very common, such as *Aspergillus* (a common mold found everywhere) and *Pleurophragmium* (a common saprophyte on woody or herbaceous plant material). We did not test for pathogenicity of any of the observed fungi, so some (i.e., *Alternaria*, *Fusarium*, and *Rhizoctonia*) could have been acting as parasitic causal agents of disease, aiding the decline in health of the ragweed plants, or as saprophytes existing on plant tissues dead from other causes. While *Rhizoctonia* are generally known as pathogens of belowground tissue, they can infect aboveground tissue (Coyier and Roane, 1986; Runion and Kelley, 1993; Runion et al., 1994a,b) or survive saprophytically on dead plant material in soil. Powdery mildew is a foliar disease of numerous plant species (Agrios, 1978) and is known to occur on common ragweed (Farr et al., 1989). We observed both the imperfect (*Oidium*) and the perfect (*Erysiphe*) states on ragweed in this study. Although pathogenicity tests were not conducted, these fungi were likely infecting ragweed leaves and aiding the declining health of the plants. Lastly, although *Phoma* were infrequently observed on ragweed leaves in this study, they also were likely acting as parasitic agents causing the leaf spot (Bohár et al., 2009) observed on several plants.

Atmospheric CO_2_ concentration had no effect on the frequency of any fungus observed on ragweed leaves and there were no significant CO_2_ by ecotype interactions (Table 2). However, ecotype did impact frequency of some of the foliar fungi. Powdery mildew was significantly higher (80%) on susceptible compared with resistant plants. Susceptible plants also showed a trend for increased frequency of *Rhizoctonia* on leaves. In contrast, *Fusarium* was observed more frequently (54%) on resistant plants, with *Pleurophragmium* showing a similar trend (Table 2).

**Table 2:** **Effects of atmospheric CO_2_ concentration (ambient, A = 375 μ mol mol^−1^; elevated, E = ambient + 200 μ mol mol^−1^) on frequency of fungi associated with two ecotypes (R = Resistant; S = Susceptible to glyphosate) of common ragweed leaves**.

**CO_**2**_ level**	**Ecotype**	***Pleurophragmium***	***Aspergillus***	***Alternaria***	***Fusarium***	**Powdery mildew^†^**	***Rhizoctonia***	***Phoma***
A	R	50.9^‡^	0	34.2	66.0	26.7	11.9	2.5
E	R	53.2	2.3	39.2	55.9	32.3	13.3	0
A	S	43.6	0	38.9	38.9	50.9	26.6	0
E	S	40.0	0	40.0	40.0	55.0	22.5	5.0
**Statistics**		***P*-values**	***P*-values**	***P*-values**	***P*-values**	***P*-values**	***P*-values**	***P*-values**
CO_2_		0.836	0.343	0.893	0.504	0.347	0.906	0.783
Ecotype		0.058	0.343	0.559	0.016	0.002	0.052	0.783
CO_2_ × Ecotype		0.516	0.343	0.859	0.599	0.780	0.694	0.191
A vs. E in R		0.545	0.191	0.826	0.385	0.389	0.845	0.450
A vs. E in S		0.750	1.000	0.975	0.945	0.630	0.718	0.261
R vs. S in A		0.317	1.000	0.772	0.033	0.012	0.095	0.450
R vs. S in E		0.075	0.191	0.590	0.128	0.023	0.228	0.261

Means with associated separation statistics are shown.

† Both the imperfect (Oidium) and perfect (Erysiphe) states of the powdery mildew fungus were observed.

‡ Data are percent of cut plant sections with which the fungus was associated.

Occurrence of fungi on plant leaves under elevated CO_2_ can be highly variable depending upon the host, microbe, and environment (Ziska and Runion, 2007). For example, Runion et al. (1994a) found that *Penicillium* decreased, *Aspergillus* remained unchanged, and total other fungi increased in the phyllosphere of cotton (*Gossypium hirsutum* L.) under elevated CO_2_. Thompson et al. (1993) found that powdery mildew (*Erysiphe graminis*) on wheat (*Triticum aestivum* L.) increased or decreased under elevated CO_2_ based on interactions between leaf N and water content. Other research has shown that plant diseases can increase (Chakraborty et al., 2000; Eastburn et al., 2010; McElrone et al., 2010), decrease (McElrone et al., 2005; Strengbom and Reich, 2006; Eastburn et al., 2010; Runion et al., 2010), or (as observed in the current study) remain unaffected (Tiedemann and Firsching, 2000; Karnosky et al., 2002; Ferrocino et al., 2013; Oehme et al., 2013) by elevated CO_2_.

All five of the fungal genera found on stems (*Aspergillus*, *Alternaria*, *Fusarium*, *Rhizoctonia*, and *Phoma*) were also observed on leaves, although *Aspergillus*, *Alternaria*, and *Phoma* were observed more frequently on stems than on leaves (Table 3). As with leaves, neither CO_2_ concentration nor its interaction with ecotype showed any significant effect on fungal frequency on ragweed stems. *Alternaria* was the only fungus significantly affected by ragweed ecotype and was observed more frequently (15%) on susceptible compared with resistant plant stems. It should be noted that, as with leaves, whether these fungi were living as pathogens or saprophytes is not known. There is very little information regarding the effects of elevated CO_2_ on fungi or fungal pathogens on plant stems and we found no information on these factors on weed stems. However, Runion et al. (2010) did find that the incidence of two stem diseases was lower when inoculated onto elevated CO_2_-grown, compared with ambient CO_2_-grown, pine seedlings; severity of both diseases was unaffected by CO_2_ concentration.

**Table 3:** **Effects of atmospheric CO_2_ concentration (ambient, A = 375 μ mol mol^−1^; elevated, E = ambient + 200 μ mol mol^−1^) on frequency of fungi associated with two ecotypes (R = Resistant; S = Susceptible to Glyphosate) of common ragweed stems**.

**CO_**2**_ level**	**Ecotype**	***Aspergillus***	***Alternaria***	***Fusarium***	***Rhizoctonia***	***Phoma***
A	R	9.3^†^	76.1	52.3	38.4	48.0
E	R	7.5	75.2	33.6	45.7	36.4
A	S	9.5	90.7	31.4	28.6	56.1
E	S	10.1	84.2	36.8	28.2	30.8
**Statistics**		***P*-values**	***P*-values**	***P*-values**	***P*-values**	***P*-values**
CO_2_		0.884	0.622	0.556	0.648	0.140
Ecotype		0.744	0.044	0.465	0.250	0.858
CO_2_ × Ecotype		0.784	0.592	0.232	0.843	0.833
A vs. E in R		0.766	0.975	0.212	0.854	0.347
A vs. E in S		0.927	0.470	0.653	0.644	0.226
R vs. S in A		0.970	0.070	0.180	0.338	0.783
R vs. S in E		0.681	0.237	0.728	0.491	0.982

Means with associated separation statistics are shown.

† Data are percent of cut plant sections with which the fungus was associated.

Unlike stems, all fungi found on roots were unique from the aboveground plant parts (Table 4). In general, fungi found on roots are saprophytes commonly found in soil (*Penicillium*, *Aureobasidium*, and *Oidiodendron*). One exception to this is *Diplodia* which can exist saprophytically or pathogenically, albeit generally associated with aboveground plant parts (Tainter and Baker, 1996); this fungus was observed on only a few root systems. The other exception to this was the presence of vesicular-arbuscular mycorrhizae (VAM), which are symbiotic associations between plant roots and fungi (Powell and Bagyaraj, 1984). Both vesicles and arbuscules were observed in ragweed roots in this study, but the VAM fungal associates were not identified. The fact that VAM frequency was unaffected by CO_2_ concentration was not unexpected as this has been previously reported (O'Neill et al., 1991; Monz et al., 1994; Runion et al., 1994a). O'Neill (1994) suggested that ectomycorrhizal infection might increase under higher levels of atmospheric CO_2_, whereas VAM might show less response, owing to a higher demand by ectomycorrhizas for plant-produced C; a trend supported by much of the available literature. However, Runion et al. (1997) argued that differences in quantification—VAM evaluations being less quantitative with only percent infection assessed—might also explain, in part, the general trend for ectomycorrhiza to show positive responses to elevated CO_2_ while VAM do not.

**Table 4:** **Effects of atmospheric CO_2_ concentration (ambient, A = 375 μ mol mol^−1^; elevated, E = ambient + 200 μ mol mol^−1^) on frequency of fungi associated with two ecotypes (R = Resistant; S = Susceptible to Glyphosate) of common ragweed roots**.

**CO_**2**_ level**	**Ecotype**	**VAM^†^**	***Penicillium***	***Aureobasidium***	***Oidiodendron***	***Diplodia***
A	R	27.8^‡^	13.9	19.8	6.7	0
E	R	38.9	2.8	11.0	1.4	2.6
A	S	24.8	20.5	17.9	5.6	0
E	S	27.1	5.3	12.9	2.5	2.6
**Statistics**		***P*-values**	***P*-values**	***P*-values**	***P*-values**	***P*-values**
CO_2_		0.805	0.019	0.389	0.169	0.118
Ecotype		0.629	0.305	0.371	0.601	0.118
CO_2_ × Ecotype		0.923	0.780	0.866	0.606	0.118
A vs. E in R		0.808	0.112	0.619	0.514	1.000
A vs. E in S		0.915	0.056	0.465	0.185	0.037
R vs. S in A		0.784	0.356	0.602	0.996	1.000
R vs. S in E		0.682	0.588	0.451	0.466	0.037

Means with associated separation statistics are shown.

† VAM are vescicular-arbuscular mycorrhizae.

‡ Data are percent of cut plant sections with which the fungus was associated.

Atmospheric CO_2_ concentration had a significant effect only on the frequency of *Penicillium* which was lower on plant roots growing under elevated CO_2_ (Table 4). Runion et al. (1994a) observed that *Penicillium* associated with cotton leaves was lower under elevated CO_2_. Neither ecotype nor their interaction with CO_2_ had any effects on fungi associated with ragweed roots. As with plant stems, there is very little information regarding the effects of elevated CO_2_ on fungi or fungal pathogens on plant roots and no information on weed roots.

The fact that herbicide susceptible ragweed plants had a higher frequency of powdery mildew, as well as several other potentially pathogenic fungi, suggests that susceptible plants might be less of a problem or easier to control as they succumb to disease. This could result in lowering herbicide rates and/or application frequency. *Fusarium* spp. are common plant pathogens, thus higher levels on herbicide resistant plants indicates an opportunity to examine its potential as a bio-control agent for these hard to control weeds. However, higher frequencies of fungi implies a greater inoculum load for future infections. If weeds harbor diseases that are able to infect crops (e.g., soybean rust on kudzu), increased frequency of disease on weeds would imply a greater threat to crops. Further, as atmospheric CO_2_ continues to rise, larger plants could withstand higher levels of infection adding to the inoculum load, as well as making the larger weeds more difficult to control.

## Conclusions

Growing awareness of herbicide resistant weed populations can alter control strategies. Further complicating this control issue is the rise in atmospheric CO_2_, which can positively increase weed growth; how elevated atmospheric CO_2_ might affect herbicide resistant weeds has not been investigated. We grew common ragweed plants that were resistant or susceptible to glyphosate under ambient and elevated CO_2_ conditions. While no significant CO_2_ by ecotype interactions were noted, main effects of both elevated atmospheric CO_2_ and glyphosate resistance increased top, root, and total biomass. Increased growth due to either ecotype or elevated CO_2_ suggests that future control strategies may need to be amended due to such complexities.

Fungi from 13 genera, several of which can either be pathogens or saprophytes (i.e., *Alternaria*, *Fusarium*, *Rhizoctonia*), were observed on ragweed. Fungal frequencies were not affected by CO_2_ concentration or its interaction with plant ecotype. The common foliar disease powdery mildew (*Oidium*, *Erysiphe*) was significantly higher on glyphosate susceptible plants. Susceptible plants also showed an increased frequency of *Rhizoctonia* on leaves and *Alternaria* on stems; however, *Fusarium* was observed more frequently on herbicide resistant ragweed leaves. The variable occurrence of fungi on ragweed ecotypes indicates that impacts on weed-crop competition and control strategies will likely also be variable and complicated; this deserves further investigation.

When a population of weeds develops resistance to herbicides, it is unknown what other traits might also be selected for or against. For example, how herbicide resistant weeds will respond to plant diseases is unknown but could have positive or negative consequences both to weed health and control and possibly to the crops with which they compete. Also, how rising atmospheric CO_2_ might affect this resistance and its associated effects on plant diseases has not been addressed. This study reports the first information on the effects of elevated CO_2_ on growth of herbicide resistant plants and on the effects of herbicide resistance and elevated CO_2_ on fungi associated with weeds. How herbicide resistance affects plant diseases and how rising atmospheric CO_2_ might impact this needs to be addressed further, not only with other important weed species but also with crops.

### Conflict of interest statement

The authors declare that the research was conducted in the absence of any commercial or financial relationships that could be construed as a potential conflict of interest.
